# Medical Schools and Degrees

**Published:** 1892-09-24

**Authors:** 


### Medical Schools and Degrees.

The time is close at hand when those young men who have not already decided what medical degree they wish to take and what hospital or school they propose to study at must come to a decision. The question of the degree had better be settled first. A man wishes to be M.D., or he will be content with the qualification of a general practitioner. But whichever he chooses he should choose at the commencement of his studies, and lay out all his plans accordingly. After the question of degree has been settled, it will be necessary to make choice of a hospital or medical school.

It is here taken for granted that two points, which are really prior to these, have been already considered and dealt with, viz., the question of money and the passing of the preliminary examination. No young man, or, at least, no ordinary young man, should decide to take a medical qualification unless he can command at least £500. Ten years ago less might have served, but now that all medical students are compelled by statute to take a course of study extending over five full years or more, it is manifest that the very smallest sum which will meet the necessities of the case is £500. Parents must also remember that no youth can be registered as a medical student until he has passed the statutory preliminary examination, and that until a student is registered any medical studies he may undertake at a hospital or elsewhere are not reckoned as part of his qualifying five years' course.

Returning to the "degree" question. There are some injudicious persons who urge every young man indiscriminately to enter for the highest degrees. They say it is always best to aim high. "Aim at the sun," say they, "and if you do not hit the sun, you will perhaps hit the moon." This advice to some young men is nonsense, and nonsense of a very injurious kind. It is obvious that many are destitute of the capacity for severe and prolonged study. They can work up to a certain point very well. But when they get beyond that point they begin to break down. Now, any man who is to fill any position above the ordinary in the medical profession must not only have the capacity to work for a high degree, but the still wider and stronger capacity to continue working with constantly increasing intellectual power and expansiveness in after life. But the majority of men cannot do this. They can fill moderate spheres with great acceptance and usefulness; wider spheres they cannot fill at all. Much of the misery which we all read of, and some of us occasionally see in connection with the higher ranks of law and medicine, is due largely to the foolish ambition of ill-judging parents who mistook the nature and extent of their son's capacities in early life.

The position of a medical man is always honourable if the man himself be honourable. If the ordinary student may not look forward to a brilliantly ambitious career he can confidently anticipate a life of the greatest possible usefulness and of as much honour as any reasonable man may desire. In advising parents and students, therefore, on the subject of degrees, we venture to suggest that very earnest consideration should be given to the two questions of the youthful student's intellectual capacity, and of the money that can be placed at his disposal.

Let us suppose that our young man is of ordinary ability and industry. He has done fairly well at school, but not exceptionally well. He has some regard for books, but is by no means an all-devouring reader. Is such a young man to be urged upon a course which the very cleverest only can pursue with safety? Or should he not rather be persuaded to strive for that medium position which is within his means and capacity, and which he will almost certainly have to fill in after life? In our judgment, the only course which is at once both wise and kind is to urge upon such a young man that he should be content with a moderate qualification. It is probable that a great many parents have entirely agreed with us up to this point, and the more so as the larger proportion of those who put their sons into medicine are persons of moderate means, and have other children's futures to think of as well as that of the youth who is ambitious to be a doctor.

For moderate abilities, then, a moderate qualification is to be sought. In England, as opposed to Scotland and Ireland, the young man who intends to be a general practitioner usually takes two distinct qualifications, and sometimes three. The membership of the Royal College of Surgeons—M.R.C.S.—he invariably takes. In addition to this he often takes, and should always take, the licentiateship of the Royal College of Physicians—L.R.C.P. Nearly all general practitioners find it desirable to become licenciates of the Apothecaries' Society, and this is a registrable qualification known as the L.S.A. With these three diplomas the English general practioner is usually content. It is open to him, however, at any time in after life to devote himself to study with the view of taking the higher qualification of M.D., and, as a matter of fact, this is often done.

Now, where does the young man generally study who enters for any or all of these qualifications? His usual place of study is a London or provincial hospital. There are eleven or twelve hospitals in London to which medical schools are attached, and a considerable number in the country. The names of all these medical schools are given in the list below. The chief point to remember is that the dean of each medical school is happy to furnish the fullest possible particulars to all parents and young men who have any personal interest in the matter. Many persons adopt the plan of writing to the Deans of all the medical schools for their regulations, and of choosing a hospital for study after a careful comparison among them all. This method may be recommended for general adoption.

There still remains for consideration the question of the higher degree of M.D. This degree carries with it many advantages, and is muc: coveted in the profession. It can only be obtained at an university, and it involves five or six years of study, two or three at least of which must be taken at the university whose M.D. degree is sought. There are a good many universities which grant medical degrees in this country; five of them are in England, four in Scotland, and two in Ireland. The young Englishman who can afford the time and the money usually prefers to study at Oxford or Cambridge, and to take the M.D. of one of those ancient universities. The man whose ambition and capacity may be as great, but whose purse is lighter, makes the M.D. of London or Edinburgh his choice. Very few Englishmen study medicine in Ireland, though Trinity College, Dublin, and the Royal University of that country give coveted degrees.

It is not for us to select for readers the university or universities which should be preferred. It is really, to a large extent, a question of money, time, and early educational advantages. The wise thing to do is to write, as in the case of the hospitals, to the Dean of the medical faculty of each university in Great Britain, and, after careful and prolonged consideration, to choose that which appears to be best suited to the means, the intellectual capacity, and the general circumstances of the student. Are there then no medical schools which stand out prominently as educational institutions of exceptional quality? There are! But of infinitely more importance than the choice of a medical school is the determination of the student to work with intelligence and conscience, and to make the best possible use of all the manifold advantages which every modern medical school in this country can so abundantly offer.

